# Advances in rapid ADAMTS13 testing for thrombotic thrombocytopenic purpura: a systematic review of diagnostic assays and the role of clinical probability tools

**DOI:** 10.3389/fmed.2026.1896867

**Published:** 2026-09-10

**Authors:** Rula Ismaiel, Alhassan AlMaqaleh, Rida Hamid Hussain, Amna M. Anis, Yahya Arabi, Sarah Albar, Muhammad Raihan Sajid, Muhammad Faisal Ikram

**Affiliations:** 1 College of Medicine, Alfaisal University, Riyadh, Saudi Arabia; 2 Department of Pathology, College of Medicine, Alfaisal University, Riyadh, Saudi Arabia; 3 Department of Anatomy, College of Medicine, Alfaisal University, Riyadh, Saudi Arabia

**Keywords:** ADAMTS13, biomarkers, diagnosis, diagnostic accuracy, thrombotic thrombocytopenia purpura (TTP)

## Abstract

**Background:**

Thrombotic thrombocytopenic purpura (TTP) is a life-threatening thrombotic microangiopathy caused by severe ADAMTS13 deficiency. This systematic review evaluates recent diagnostic advances, focusing on rapid assays, clinical prediction tools, and emerging biomarkers.

**Methods:**

Following PRISMA 2020 guidelines, we searched PubMed, Embase, Cochrane Library, Google Scholar, and ClinicalTrials.gov (2015–2026). We included studies evaluating ADAMTS13-based or adjunct biomarkers for TTP diagnosis, reporting diagnostic accuracy or turnaround time. Risk of bias was assessed using QUADAS-2.

**Results:**

From 2,217 records and 42 registry entries, 14 studies met inclusion criteria. ADAMTS13 activity <10% remained the key diagnostic threshold. Rapid automated platforms reduced turnaround time from >72 h to 17–35 min.

**Key findings include:**

HemosIL AcuStar assay (100% sensitivity, 94.6%–98.9% specificity, 33-min turnaround); CLEIA (17-min turnaround, near-perfect agreement with reference methods, *κ* = 0.95–0.96); bedside Technoscreen assay (100% sensitivity, 90% specificity, 10-min result). Clinical prediction tools demonstrated variable performance across settings, with PLASMIC showing the most favourable balance of sensitivity and specificity (pooled sensitivity 0.85, specificity 0.89 at the conventional threshold of ≥6). Meta-analysis and multicentre data indicate that performance is influenced by patient age, laboratory parameters, and the chosen cut-off. Open-conformation ADAMTS13 assays detected abnormal conformation in 97% of acute iTTP samples, supporting relapse monitoring.

**Conclusion:**

TTP diagnosis is shifting from slow reference-laboratory confirmation toward an integrated strategy combining rapid automated ADAMTS13 testing, clinical probability scoring, and adjunct biomarkers. This approach enables earlier diagnosis, faster treatment initiation, and improved relapse surveillance.

**Systematic Review Registration**: identifier CRD420261363424.

## 1 Introduction

### 1.1 Background

Thrombotic thrombocytopenic purpura (TTP) is a rare and potentially fatal thrombotic microangiopathy (TMA). It is characterized by microangiopathic hemolytic anemia (MAHA) and severe thrombocytopenia, and may cause fever, neurologic manifestations, and other organ ischemia; the historical pentad is present in only a minority of patients at diagnosis (1, 2). In 2001, researchers discovered that a severe deficiency of von Willebrand factor-cleaving protease (VWF-CP), also known as ADAMTS13 (a disintegrin and metalloproteinase with thrombospondin type 1 motifs, member 13), is the primary mechanism underlying TTP (3–5). This deficiency leads to accumulation of ultra-large von Willebrand factor (VWF) multimers, causing uncontrolled platelet aggregation and microvascular thrombosis (6, 7). Severe ADAMTS13 deficiency can be inherited (congenital TTP, cTTP, also known as Upshaw-Schulman syndrome) or, more commonly, acquired due to anti-ADAMTS13 autoantibodies (immune-mediated TTP, iTTP) (1, 8). Biallelic ADAMTS13 gene mutations on chromosome 9q34 cause autosomal recessive cTTP (9). iTTP accounts for over 90% of cases and typically presents in adulthood, while cTTP often appears before age ten or during pregnancy (10, 11). ADAMTS13 cleaves ultra-large VWF multimers released from endothelium and platelets into smaller, less adhesive forms (12, 13), and both inhibitory autoantibodies and antigen depletion contribute to its deficiency (14, 15).

### 1.2 Importance of rapid diagnosis

Severe ADAMTS13 deficiency (<10%–20% activity in acute cases) and the presence of inhibitory autoantibodies in iTTP are essential for diagnosis (7, 16, 17). Standard laboratory tests measure platelet count, hemoglobin, and schistocytes on blood smears. Distinguishing iTTP from cTTP requires detection of ADAMTS13 autoantibodies during an acute episode or recovery of ADAMTS13 activity to >10%–20% in remission. ADAMTS13 activity <10% is diagnostic for TTP. Inhibitor assays differentiate iTTP from cTTP (3). Genetic sequencing may be necessary for cTTP diagnosis, as not all iTTP patients have detectable autoantibodies, and some do not restore ADAMTS13 activity in remission (1, 18, 19). TTP was consistently fatal when first described (20). Over the past decades, the fatality rate has dropped from over 90% to less than 15% as acute care has improved (21, 22). Contemporary cohorts confirm this decline, with recent registry and multicentre data further characterising mortality and fatal outcomes in the caplacizumab era (23, 24). However, because ADAMTS13 test results are often not immediately available, empirical treatment is frequently started based on clinical suspicion. The primary goal in evaluating a patient with suspected TTP is to initiate plasma exchange as soon as possible (25). A secondary goal is to avoid unnecessary catheter insertion and plasma exchange in patients with other TMAs who may benefit from different treatments (16, 26). Therapeutic plasma exchange remains the foundation of treatment, since it removes autoantibodies and restores ADAMTS13, and is begun as soon as the diagnosis is suspected (10, 22). Immunosuppression with corticosteroids and rituximab, and the anti-VWF nanobody caplacizumab, are established adjuncts (27, 28). Because delay increases morbidity and mortality, plasma exchange should not be postponed while awaiting confirmatory ADAMTS13 activity results. A major obstacle to timely TTP diagnosis is that not all hospitals have rapid access to ADAMTS13 testing, with results often taking two to five days. The FRETS (fluorescence resonance energy transfer) assay is the gold standard for evaluating ADAMTS13 activity, while activity ELISA is another common method (16, 21). Chromogenic ELISA and FRETS-VWF73 are newer assays that provide faster results while reducing manual challenges (29).

### 1.3 Emerging diagnostic advances

>Recent diagnostic advances focus on improving ADAMTS13 activity assessment, which remains central to confirming TTP. Severe ADAMTS13 deficiency (<10%) is highly specific for TTP and enables differentiation from other TMAs (7). Established assays such as FRETS-VWF73 provide direct, reliable measurement of ADAMTS13 enzymatic activity (30, 31). However, variability between assay methodologies and incomplete standardization across laboratories can lead to differing reported activity levels (30, 32). These limitations have driven the development of automated assays, rapid testing platforms, and biomarker-based strategies aimed at improving standardization, reproducibility, and diagnostic performance. Collectively, these technologies aim to make TTP diagnosis faster, more accurate, and better integrated into clinical decision-making.

## 2 Systematic review objectives

This systematic review aims to synthesize existing evidence on diagnostic approaches for TTP. Primary research question: Do ADAMTS13-based and adjunct biomarkers improve TTP diagnosis compared to standard methods?

## 3 Methods

### 3.1 Information sources and search strategies

We conducted this systematic review in accordance with PRISMA 2020 guidelines. We structured the review using the PICO framework:

Population: Adults with suspected or confirmed TTP
Intervention: ADAMTS13-based and adjunct biomarkers
Comparison: Standard diagnostic pathways
Outcomes: Diagnostic accuracy, time to diagnosis, clinical decision-making

The review was registered on https://www.crd.york.ac.uk/PROSPERO/view/CRD420261363424, PROSPERO (ID: CRD420261363424).

We searched PubMed, Embase, Cochrane Library, Google Scholar, and ClinicalTrials.gov for publications from 2015 to 2026. Main keywords included TTP, ADAMTS13, biomarkers, diagnosis, diagnostic accuracy, and rapid testing. MeSH terms and Boolean operators were applied where applicable.

### 3.2 Eligibility criteria

Inclusion Criteria: We included peer-reviewed, full-text English studies published from 2015 onward that evaluated adults (≥18 years) with suspected or confirmed TTP, or mixed TMA populations from which TTP-specific data could be extracted. Eligible index tests were ADAMTS13 activity by any method, antigen and anti-ADAMTS13 antibody assays, adjunct laboratory markers, point-of-care tests, and diagnostic scores or algorithms. Eligible designs were randomized trials, prospective or retrospective cohorts, case-control and cross-sectional diagnostic-accuracy studies, and case series of at least ten patients. Primary outcomes were diagnostic accuracy, turnaround time, and impact on treatment decisions; secondary outcomes included biomarker correlation with severity, differentiation from other TMAs, and inter-laboratory variability

Exclusion Criteria: We excluded, exclusively pediatric, animal, or *in-vitro* studies; isolated congenital TTP without acquired data; other TMAs without a direct TTP comparison; purely therapeutic or basic-science studies without a diagnostic component; VWF multimer analysis without ADAMTS13 assessment; and studies without extractable diagnostic-performance data. Also excluded: conference abstracts, letters, editorials, commentaries, non-systematic narrative reviews, studies with insufficient methodological detail for quality assessment, duplicate publications or overlapping cohorts (prioritizing most comprehensive/recent data), non-peer-reviewed literature (preprints, white papers, theses), retracted articles, papers unavailable in full text or in English.

### 3.3 Study selection and screening progress

Two investigators independently screened titles and abstracts using Rayyan AI, resolving conflicts by consensus. Standardized data extraction included study characteristics, diagnostic tests and biomarkers evaluated, cutoff values, accuracy measures (sensitivity, specificity, PPV, NPV), turnaround time, and clinical impact on decision-making.

### 3.4 Risk of bias assessment

Risk of bias was assessed with the QUADAS-2 tool, which appraises four domains, patient selection, index test, reference standard, and flow and timing, each rated as low, high, or unclear risk.

QUADAS-2 was performed by one investigator and checked by another.

The complete methodological quality and risk of bias assessment, evaluated using the Quality Assessment of Diagnostic Accuracy Studies (QUADAS-2) tool, is detailed in Table 1.

**Table 1:** Risk of bias assessment using the QUADAS-2 tool.

Study	Risk of bias				Applicability concerns		
	Patient selection	index test	Reference standard	Flow & timing	Patient selection	Index test	Reference standard
Coelho (2020)	High	Unclear	Low	Low	High	Low	Low
Mancini (2019)	Low	Low	Low	Low	Low	Low	Low
Park (2020)	Low	Low	Low	Low	Low	Low	Low
Liu (2020)	Low	Low	Low	Low	Low	Low	Low
Miyazaki (2021)	Low	Low	Low	Low	Low	Low	Low
Stephenson (2023)	Low	Low	Low	Low	Low	Low	Low
Dimopoulos (2022)	Low	Low	Low	Low	Low	Low	Low
Herrera Rivera (2023)	Low	Low	Low	Low	Low	Low	Low
Kubo (2025)	High	Low	Low	Low	High	Low	Low
Chen (2025)	Low	Low	Low	Low	Low	Low	Low
Lv (2025)	Low	Low	Low	Low	Low	Low	Low
Yong (2024)	Low	Low	Low	Low	Low	Low	Low
Bonnez (2024)	High	Low	Low	Low	High	Low	Low
Joly (2025)	Low	Low	Low	Low	Low	Low	Low

### 3.5 Data synthesis

Due to anticipated heterogeneity, we performed a narrative synthesis. Findings were grouped into four categories: ADAMTS13 assays, adjunct biomarkers, novel diagnostics, and comparative diagnostic strategies.

A meta-analysis was not performed because of substantial clinical and methodological heterogeneity across the included studies in their populations, index-test methods, diagnostic thresholds, reference standards, reported outcomes, and study designs, which would make any pooled estimate unstable and potentially misleading; we therefore judged a structured narrative synthesis to be the appropriate approach.

## 4 Results

### 4.1 Study selection

**Figure 1:**
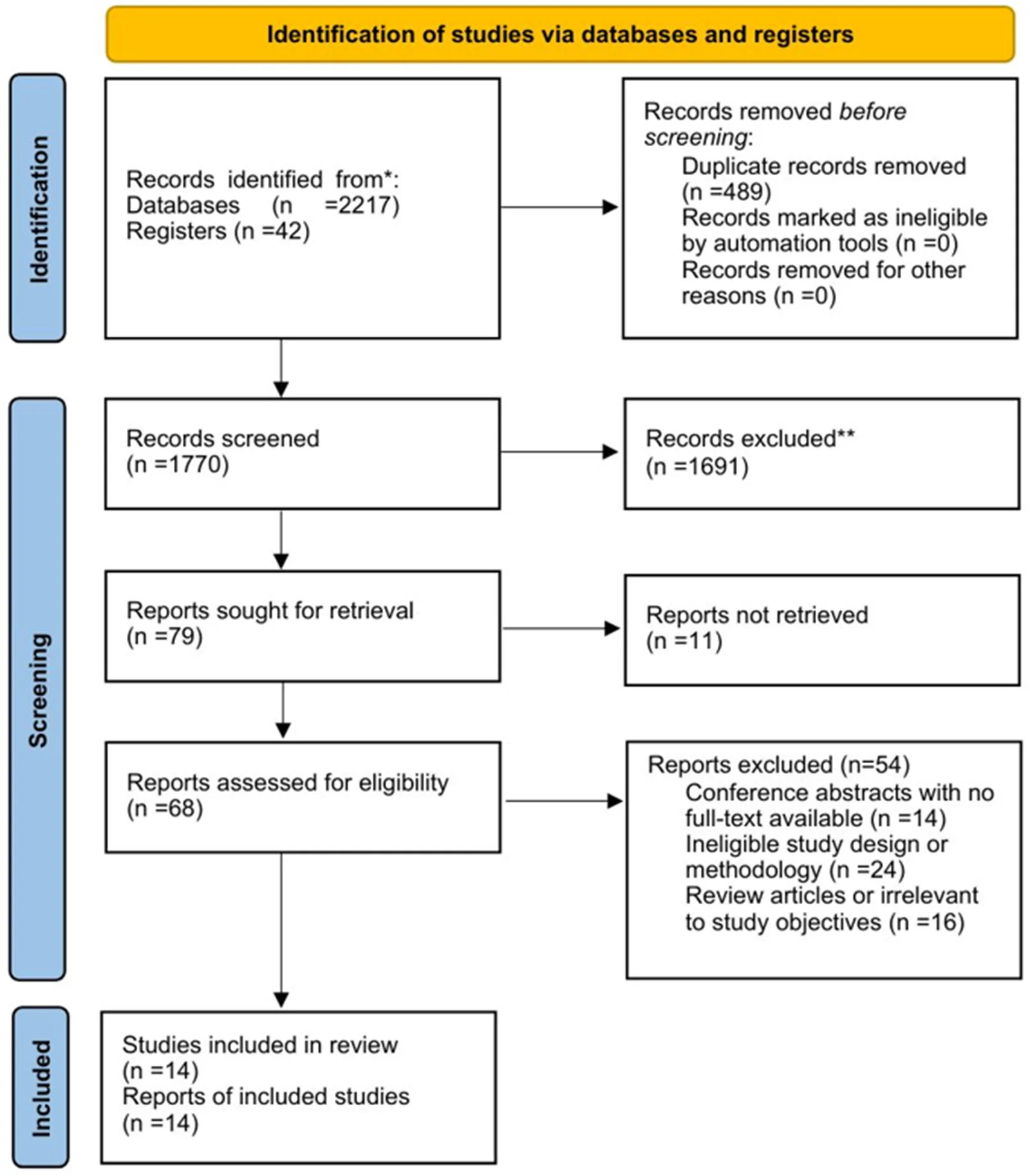
PRISMA 2020 flow diagram of the study selection process.

Our initial electronic search from five databases (PubMed, Cochrane Library, Google Scholar, Embase, and various governmental websites) yielded 2,217 records and 42 registry entries. After removing 489 duplicate studies, 1,770 articles remained for title and abstract screening which was done using Rayyan AI. After the initial pass through 68 studies remained for an in-depth full-text assessment from which 14 studies met the eligibility criteria and were included in the final review. The PRISMA 2020 flow diagram (Figure 1), recorded the identification screening, and inclusion of studies for this paper. The screening process was double-blind, with two investigators screening independently to reduce bias.

### 4.2 Study characteristics

A comprehensive summary of the included study characteristics is presented in Table 2.

**Table 2:** Key characteristics of the reviewed studies.

Study	Aim of study	No. of participants	Participating cohorts	Main findings
Kubo (2025) (33)	To evaluate a novel automated CLEIA for rapid ADAMTS13 activity and inhibitor measurement	158	iTTP (acute/remission), other TMAs, and healthy controls	The study validated a novel, fully automated chemiluminescent enzyme immunoassay (CLEIA) that accurately measures ADAMTS-13 activity and inhibitory autoantibodies in just 17 min. The assay demonstrated high diagnostic performance and a strong correlation with conventional manual methods, enabling rapid in-hospital diagnosis and effective follow-up monitoring for TTP patients.
Chen (2025) (34)	To investigate the diagnostic utility of the LDH-to-serum creatinine (LDH/sCr) ratio in identifying TTP	61	Patients with suspected TTP tested for ADAMTS13 activity	The study found that the newly developed LCRP algorithm (incorporating the lactate dehydrogenase-to-serum creatinine ratio, reticulocyte percentage, and platelet count) significantly outperformed the standard PLASMIC scoring model in accurately identifying Chinese patients with thrombotic thrombocytopenic purpura.
Lv (2025) (35)	To differentiate critically ill patients with TTP from TTP-like syndrome using a novel predictive model	86	Patients with TTP and TTP-like syndrome	This study found that thrombotic thrombocytopenic purpura (TTP) and TTP-like syndrome are distinct diseases with different clinical characteristics and treatment responses, and it successfully developed a new five-variable predictive model with 96.9% accuracy to differentiate between them.
Yong (2024) (36)	To provide practical considerations and clinical validation for the rapid AcuStar ADAMTS13 assay	40	Patients with suspected acute TMA	While the rapid AcuStar® ADAMTS13 assay correlates well with traditional ELISA methods in patients with a high clinical suspicion of acute TTP, it can produce false-positive results in approximately 2% of cases with low clinical suspicion, particularly those with concurrent conditions like sepsis or cancer.
Bonnez (2024) (37)	To develop and validate automated FO-SPR immunoassays for ADAMTS13 antigen and conformation evaluation	108	iTTP (acute/remission), other TMAs, and healthy controls	This study successfully developed automated, easy-to-use fiber optic surface plasmon resonance (FO-SPR) assays for measuring ADAMTS-13 antigen and conformation, demonstrating that these assays have high analytical sensitivity and strong correlation with traditional ELISA reference methods for diagnosing immune-mediated thrombotic thrombocytopenic purpura (iTTP).
Joly (2025) (38)	To conduct a real-world evaluation of the automated ADAMTS13 activity method on the Ceveron s100 analyzer	344	Patients with suspected TMA (prospective and retrospective cohorts)	The Technofluor assay on the Ceveron s100 analyzer is a rapid and reliable automated method that demonstrates excellent analytical and clinical performance for the diagnosis and monitoring of thrombotic thrombocytopenic purpura (TTP) in real-world conditions.
Stephenson (2023) (39)	To evaluate the Technoscreen semi-quantitative screening test for rapid TTP diagnosis and exclusion	102	Patients with suspected TMA and healthy controls	The Technoscreen assay is a highly sensitive screening tool (100% sensitivity and NPV) that reliably excludes TTP diagnosis in routine clinical practice. However, it is prone to significant quality issues, including high false-positive rates and batch inconsistencies, necessitating that all positive results be confirmed by quantitative testing.
Dimopoulos (2022) (40)	To compare the performance of the automated HemosIL method against the standard FRET assay	706	Consecutive plasma samples, including 212 diagnostic samples	The study concludes that the rapid, automated HemosIL method for quantifying ADAMTS13 activity is highly comparable to the standard FRET method, providing excellent clinical accuracy for the real-time diagnosis of thrombotic thrombocytopenic purpura.
Herrera Rivera (2023) (41)	To develop a machine learning algorithm to reduce overutilization of ADAMTS13 testing and assist in stewardship	281	Patients for whom ADAMTS13 activity testing was ordered	The study successfully developed a machine learning decision tree algorithm using PLASMIC score features that achieved 81% overall accuracy and a 100% negative predictive value, providing a reliable tool to rule out thrombotic thrombocytopenic purpura (TTP) and reduce laboratory test overutilization.
Zhao (2020) (42)	To validate a modified PLASMIC score including the LDH/ULN ratio in a Chinese TTP cohort	38	Patients with suspected TTP	This study found that a modified PLASMIC score incorporating the lactate dehydrogenase/upper limit of normal (LDH/ULN) ratio identifies Chinese patients with thrombotic thrombocytopenic purpura (TTP) more accurately than the original score.
Moosavi (2020) (43)	To validate the PLASMIC score at a large multi-institutional academic medical center	46	Patients evaluated for or receiving plasma exchange for suspected TTP	While this study validated the PLASMIC score with findings similar to other institutions, the authors concluded that it provided insufficient evidence to recommend using the score alone to rule out thrombotic thrombocytopenic purpura (TTP), instead reinforcing the necessity of early ADAMTS13 testing for diagnostic triage.
Pascual (2021) (44)	To conduct a multicentric evaluation of the HemosIL Acustar chemiluminescence ADAMTS13 activity assay	163	Patients with suspected TTP or other TMAs across 10 centers	The study found that the new HemosIL AcuStar® assay is highly sensitive for detecting severe ADAMTS13 deficiency (<10% and <5%) with no false negatives, though it tends to underestimate activity levels in higher ranges and may be affected by the presence of sepsis.
Roose (2020) (5)	To evaluate if “open” ADAMTS13 conformation serves as a biomarker for subclinical iTTP	314	iTTP (acute/remission), other TMAs, and healthy donors	This study found that anti-ADAMTS13 autoantibodies from iTTP patients induce an open ADAMTS13 conformation, which serves as a novel and sensitive biomarker for detecting subclinical immune-mediated thrombotic thrombocytopenic purpura (iTTP) during remission.
Coelho (2020) (45)	To compare the diagnostic accuracy of the PLASMIC, Bentley, and French clinical prediction scores	47	Consecutive patients with clinical suspicion of TMA	In this comparative study, the PLASMIC score was identified as the most efficient and balanced clinical prediction tool for severe ADAMTS13 deficiency due to its high sensitivity, specificity, and ease of use in emergency settings compared to the Bentley and French scores.

### 4.3 Population and demographics

The included studies represent a diverse set of international evidence, seven studies were conducted in Europe (including Portugal, Denmark and the UK), four in Asia (China and Japan), two in North America (United States), and one in Australia. Retrospective designs were most prevalent and accounted for much of the diagnostic accuracy data.

### 4.4 Risk of bias discussion

Quality assessment using QUADAS-2 criteria indicated a generally low risk of bias across most domains for many studies. However, High Risk for patient selection and applicability concerns was noted in four specific studies (Coelho 2020, Kubo 2025, and Bonnez 2024) (45–37). All studies were considered to have a low risk of bias regarding the reference standard domain.

### 4.5 Performance of ADAMTS13 based assays

Multiple rapid and automated immunological assays were evaluated using reference standards to determine their diagnostic capabilities for TTP.

Table 3 provides an overview of ADAMTS13 assay methods and characteristics.

**Table 3:** Overview of ADAMTS13 assay methods and characteristics.

No.	Diagnostic method evaluated	Sensitivity	Specificity	PPV NPV *respectively*	TAT	Main findings
1	Technoscreen ADAMTS13 activity semi-quantitative screening assay	100%	90%	77% 100%	Often <72 h target manually read within 10 min.	Demonstrated 100% sensitivity for rapid TTP exclusion at the bedside. However, significant inter-operator variability and batch inconsistencies were noted, leading to a high false-positive rate (PPV 77%). Recommended strictly as a “rule-out” screening tool requiring quantitative confirmation.
2	Fully automated chemiluminescent enzyme immunoassay (CLEIA)	NR*	NR*	NR*	17 min	The study validated a novel, fully automated CLEIA that accurately measures ADAMTS-13 activity and inhibitory autoantibodies in just 17 min. The assay demonstrated high diagnostic performance and a strong correlation with conventional manual methods FRET and ELISA (*κ* = 0.96 and 0.95 respectively), enabling rapid in-hospital diagnosis and effective follow-up monitoring for TTP patients.
3	Fiber optic surface plasmon resonance (FO-SPR) immunoassays	NR*	NR*	NR*	NR	This study successfully developed easy-to-use FO-SPR assays for measuring ADAMTS-13 antigen and conformation, showing that these assays have high analytical sensitivity and strong correlation (*r* = 0.92) with traditional ELISA reference methods for diagnosing iTTP.
4	Technofluor ADAMTS-13 activity assay (Ceveron s100)	100% (at 10 IU/dL)	100% (at 20 IU/dL)	100% (at 10 IU/dL) 98% (at 20 IU/dL)	Rapid	The Technofluor assay on the Ceveron s100 analyzer is a rapid and reliable automated method that demonstrates excellent analytical and clinical performance for the diagnosis and monitoring of TTP in real-world conditions.
5	ADAMTS13 conformation ELISA (1C4-ELISA).	**96%** (low activity)/**38%** (restored activity)	**97%** for acute iTTP	*Not Explicitly sated* in healthy donors **97%** in acute iTTP samples (open conformation for ADAMTS13) **100%** healthy & **62%** remission samples closed	NR	This study found that anti-ADAMTS13 autoantibodies from iTTP patients induce an open ADAMTS13 conformation, which serves as a novel and sensitive biomarker for detecting subclinical iTTP during remission.
6	HemosIL AcuStar® ADAMTS13 activity chemiluminescent immunoassay.	100% for both the <10% and <5% cut-off values.	94.6% for the <10% cut off value 97.4% for the <5% cut-off value	84.8% → <10% 91.1% → <5% 100% for both the <10% and <5% cut off value.	33 min	The study found that the new HemosIL AcuStar® assay is highly sensitive for detecting severe ADAMTS13 deficiency (<10% and <5%) with no false negatives, though it tends to underestimate activity levels in higher ranges and may be affected by the presence of sepsis.
7	HemosIL AcuStar (Automated Chemiluminescence) vs. FRET	HemosIL: 100%, FRET: 96.4%	HemosIL: 98.9%, FRET: 98.9%	HemosIL: 93.3%, FRET: 93.1% HemosIL: 100%, FRET: 99.5%	HemosIL: 33 min, FRET: ∼1 working day	The study concludes that the rapid, automated HemosIL method for quantifying ADAMTS13 activity is highly comparable to the standard FRET method, providing excellent clinical accuracy for the real-time diagnosis of thrombotic thrombocytopenic purpura.
8	HemosIL AcuStar® (rapid assay)	NR	NR	NR	Within 30 min	While the rapid AcuStar® ADAMTS13 assay correlates well with traditional ELISA methods in patients with a high clinical suspicion of acute TTP, it can produce false-positive results in approximately 2% of cases with low clinical suspicion, particularly those with concurrent conditions like sepsis or cancer.

PPV, positive predictive value; NPV, negative predictive value; TAT, turn around time.

* See the “Main Findings” column for additional details and specific study notes.

#### 4.5.1 Technoscreen ADAMTS13 activity semi-quantitative screening assay

One study evaluated the Technoscreen ADAMTS13 activity semi-quantitative screening assay against quantitative reference assays, including ELISA (Technozym) and chemiluminescence-based AcuStar testing. Using a cutoff of <10 IU/dL for a positive result and ≥10 IU/dL for a negative result, the assay demonstrated a sensitivity of 100% [95% confidence interval (CI), 84%–100%], specificity of 90% (95% CI, 80%–95%), positive predictive value of 77% (95% CI, 58%–89%), and negative predictive value of 100% (95% CI, 93%–100%). The assay could be read manually within 10 min and was reported to support rapid decisions regarding initiation of therapeutic plasma exchange (TPE) and caplacizumab while helping avoid unnecessary treatment when TTP was excluded (36).

#### 4.5.2 HemosIL acuStar chemiluminescent immunoassay

Three studies evaluated the HemosIL AcuStar rapid chemiluminescent assay. In one study comparing the assay with an in-house FRETS-VWF73 assay and 2 commercial ELISA assays, the HemosIL AcuStar demonstrated sensitivity of 100% at both the <10% and <5% cutoffs, specificity of 94.6% at the <10% cutoff and 97.4% at the <5% cutoff, positive predictive values of 84.8% and 91.1%, respectively, and negative predictive values of 100% at both thresholds. The analytical turnaround time was 33 min. The study also noted a tendency to underestimate ADAMTS13 activity at higher activity ranges (>40%) and reported a small number of false-positive result (38).

In another comparison against an in-house FRET assay, the HemosIL AcuStar showed sensitivity of 100%, specificity of 98.9%, positive predictive value of 93.3%, and negative predictive value of 100% at a cutoff of <0.10 kIU/L (10%). Its turnaround time was 33 min compared with approximately 1 working day for the FRET assay (34). A third study comparing the rapid AcuStar assay with Technozym ELISA reported a turnaround time of within 30 min, however, sensitivity and specificity values were not reported (36).

#### 4.5.3 Other ADAMTS13-based assays

Additional ADAMTS13-based assays were also evaluated. A fully automated chemiluminescent enzyme immunoassay (CLEIA), referenced against in-house FRETS-VWF73 and commercial ELISA methods, used cutoffs of 10 IU/dL for activity and 0.5 BU/mL for inhibitor detection and had a reported turnaround time of 17 min. Although diagnostic accuracy measures were not reported in the extracted dataset, the assay was described as enabling rapid in-hospital diagnosis and follow-up of TTP as well as detection of inhibitory autoantibodies (33).

The Technofluor ADAMTS13 activity assay (Ceveron s100), evaluated against a FRETS-VWF73 reference standard, used a cutoff of 10 IU/dL for diagnosis and 20 IU/dL for follow-up. It demonstrated sensitivity of 1.0 at 10 IU/dL, specificity of 1.0 at 20 IU/dL, positive predictive value of 1.0 at 10 IU/dL, and negative predictive value of 0.98 at 20 IU/dL. The assay was described as rapid and effective for diagnosis and monitoring of TTP (38).

A fiber optic surface plasmon resonance (FO-SPR) immunoassay was compared with a 1C4 open/closed ELISA. Although sensitivity and specificity values were not reported, the assay was described as showing strong correlation with ELISA and as having potential for automated and accessible diagnosis of immune-mediated TTP (37). In addition, one study evaluated an ADAMTS13 conformation ELISA (1C4-ELISA). Open conformation was reported as a sensitive biomarker for subclinical iTTP, being detected in 96% of samples with ADAMTS13 activity <50% and in 97% (30/31) of acute iTTP samples, whereas none of the healthy donor samples showed open conformation. This assay was proposed as a tool that may help guide monitoring during remission and assess response to preemptive treatment (5).

### 4.6 Adjunct biomarkers and clinical prediction tools

#### 4.6.1 LCRP algorithm

One study evaluated the LDH/sCr ratio, Reticulocyte Percentage, Platelet Count (LCRP) algorithm, which incorporated the lactate dehydrogenase/serum creatinine ratio, reticulocyte percentage, and platelet count, using ADAMTS13 activity <10% as the reference standard. At a cutoff value of 6.7, the algorithm demonstrated sensitivity of 96.3%, specificity of 88.2%, positive predictive value of 86.7%, and negative predictive value of 96.8%. This finding derives from a single cohort (27 TTP patients) and requires validation in larger, multicentre populations before broader conclusions can be drawn (34).

### 4.7 Clinical prediction scores

Table 4 provides an overview of the validated clinical prediction tools.

**Table 4:** Overview of validated clinical prediction tools.

No.	Diagnostic method evaluated	Sensitivity	Specificity	PPV	TAT	Main findings
				NPV		
				*Respectively*		
9	Plasmic score, Bentley score, French score	PLASMIC score: 80.6%. Bentley score: 44.4%. French score: 83.3%.	PLASMIC score: 78.4%. Bentley score: 94.3%. French score: 36.2%.	PLASMIC score: 56.8% Bentley score: 80.0%.10 French score: 33.3%. PLASMIC score: 92.0%. Bentley score: 76.7%. French score: 85.0%.	Rapid	In this comparative study, the PLASMIC score was identified as the most efficient and balanced clinical prediction tool for severe ADAMTS13 deficiency due to its high sensitivity, specificity, and ease of use in emergency settings compared to the Bentley and French scores.
10	The original PLASMIC score and a modified PLASMIC score model (LDH/ULN).	Original PLASMIC score: 100%. Modified model: 100%. LDH/ULN ratio alone (≥1.87): 100%.	Original PLASMIC score: 9.5%. Modified model 95.2%. LDH/ULN ratio alone (≥1.87): 76.2%.	Original PLASMIC score: 47.22% 100% Modified model 94.6% 100%. LDH/ULN ratio alone (≥1.87): Not explicitly stated for both.	Rapid	This study found that a modified PLASMIC score incorporating the lactate dehydrogenase/upper limit of normal (LDH/ULN) ratio identifies Chinese patients with TTP more accurately than the original score.
11	The PLASMIC score	78%	63%	75% 67%	Rapid	While this study validated the PLASMIC score with findings similar to other institutions, the authors concluded that it provided insufficient evidence to recommend using the score alone to rule out TTP, instead reinforcing the necessity of early ADAMTS13 testing for diagnostic triage.
12	Machine learning decision tree model	100%	69%	67% 100%	N/A (mentions >72 h for reference labs)	The study successfully developed a machine learning decision tree algorithm using PLASMIC score features that achieved 81% overall accuracy and a 100% negative predictive value, providing a reliable tool to rule out TTP and reduce laboratory test overutilization.
13	Novel predictive model	100%	65%	78.13% 100%	rapid	This study found that TTP and TTP-like syndrome are distinct diseases with different clinical characteristics and treatment responses, and it successfully developed a new five-variable predictive model with 96.9% accuracy to differentiate between them.

#### 4.7.1 PLASMIC, bentley, and French scores

One study compared the PLASMIC, Bentley, and French clinical prediction scores using ADAMTS13 activity <10% as the reference standard. For high-risk thresholds, defined as >5 points for the PLASMIC score, >30 points for the Bentley score, and 2–3 points for the French score, sensitivities were 80.6%, 44.4%, and 83.3%, respectively. Specificities were 78.4%, 94.3%, and 36.2%, respectively. Positive predictive values were 56.8%, 80.0%, and 33.3%, and negative predictive values were 92.0%, 76.7%, and 85.0%, respectively (45). These findings suggest substantial variability in diagnostic performance across commonly used clinical prediction tools.

#### 4.7.2 Modified PLASMIC-based models

One small single-centre Chinese study (Zhao et al., 2020) evaluated a modified four-variable model (platelet count, reticulocyte percentage, indirect bilirubin, and LDH/ULN ratio) against the original PLASMIC score. Both demonstrated 100% sensitivity; specificity was 95.2% for the modified model vs. 9.5% for the original score using a non-standard cut-off. However, this result has not been externally validated and should be interpreted with caution (42). Broader validation evidence, including a meta-analysis of 13 studies (Paydary et al.), indicates that PLASMIC performs within a plausible range at the conventional threshold of ≥6, with pooled specificity of 0.89 (46).

A separate study evaluating the PLASMIC score alone reported sensitivity of 78%, specificity of 63%, positive predictive value of 75%, and negative predictive value of 67% using ADAMTS13 activity <10% as the reference standard. The same study noted that reference laboratory turnaround times ranged from several hours to several days, whereas an alternative in-house assay had a turnaround time of 30–60 min. All 46 patients initially received TPE, and treatment was discontinued in 18 of 19 patients found to have ADAMTS13 activity >10% (43).

#### 4.7.3 Machine learning and novel predictive models

Additional prediction approaches were also identified. A machine learning decision tree model using ADAMTS13 activity <10% as the target threshold demonstrated sensitivity of 100%, specificity of 69%, positive predictive value of 67%, and negative predictive value of 100%, and was proposed as a method to reduce overuse of ADAMTS13 testing while supporting clinical decision-making (41). Another study described a novel predictive model incorporating reticulocyte percentage, platelet count, schistocyte percentage, LDH/ULN, and indirect bilirubin. Using ADAMTS13 activity <10% with positive inhibitors as the reference standard and a cutoff of 3.0, the model demonstrated sensitivity of 100%, specificity of 65%, positive predictive value of 78.13%, and negative predictive value of 100%, with reported rapid application in clinical practice (35).

A recent multicentre analysis revisiting the PLASMIC score (47) demonstrated that its diagnostic performance is not uniform across settings and is materially influenced by patient age, mean corpuscular volume, and LDH, with reduced specificity in older patients and in those with confounding causes of thrombocytopenia. This large, multi-institution evidence tempers enthusiasm for score-only triage and reinforces the need for local calibration before adoption.

## 5 Discussion

### 5.1 Summary of Key findings

TTP is a medical emergency in which delayed diagnosis leads to organ injury or death. Across the included studies, rapid and automated ADAMTS13 assays shortened turnaround time from days to approximately 35 min compared with conventional ELISA or referral-laboratory testing (10).

Automated and quantitative ADAMTS13 assays showed strong agreement with reference standards such as FRETS-VWF73 and high sensitivity and specificity in acute TTP. Fluorescence-based and chemiluminescent platforms demonstrated excellent discrimination for severe ADAMTS13 deficiency (<10 IU/dL) (33, 38).

Adjunct biomarkers added value: the LDH/sCr ratio achieved an AUC of 0.895, indicating that biomarker combinations may capture disease physiology better than single laboratory values (34). The ADAMTS13 conformation assay and inhibitory antibody detection were particularly promising for equivocal cases (activity 10–20 IU/dL), where interpretation is difficult and diagnostic uncertainty has significant consequences (37).

Automated assay results agreed with conventional reference methods across several validation studies and larger sample sets, with high sensitivity, specificity, and predictive values overall (38). Minor analytical bias at certain concentration ranges matters less than an assay's ability to detect severe deficiency and guide emergency decisions. Qualitative rapid tests performed well, although lot-to-lot and operator variability in the Technoscreen assay shows that quality control remains essential even for simple platforms (48).

This regional experience should be read alongside the foundational international multicentre validation of the same platform by Moore et al. (51), which assessed 220 patients with suspected thrombotic microangiopathy across three reference centres against ELISA and in-house FRETS-VWF73, and which remains the benchmark for screening reliability and inter-operator reproducibility. Taken together, these data frame the central outstanding question of how close the field now is to universal standardization. Despite consensus frameworks (16, 18) and a WHO international standard, ADAMTS13 results are not yet fully interchangeable across platforms, since calibration, units, and thresholds still differ and lot-to-lot variability persists; genuine harmonisation will require commutable reference materials and sustained external quality assessment.

### 5.2 Interpretation of diagnostic advancements

Automated chemiluminescent (CLEIA) and fluorescence assays provide faster in-house ADAMTS13 measurement with reliability comparable to reference standards and high sensitivity for severe deficiency, which makes them valuable in acute care, whereas qualitative tools such as Technoscreen give an immediate indication while quantitative confirmation is awaited (48).

The LDH/sCr ratio, in a single study, improved discrimination when combined with platelet count, reticulocyte percentage, and standard hemolysis markers, but this requires validation. Composite algorithms showed improved discrimination compared with single laboratory parameters and traditional clinical scores in the studies that evaluated them, though these findings are based on limited evidence (34, 49). While clinical scoring systems like PLASMIC remain useful because they combine laboratory and bedside information into probability estimates, their performance varies across settings and is influenced by the chosen cut-off. The data suggest that an integrated diagnostic pathway combining quantitative ADAMTS13 testing, rapid screening tools, biomarker ratios, and clinical probability scores is a rational, testable strategy rather than an evidence-based recommendation, as no included study directly compared integrated vs. single-test approaches.

### 5.3 Validation, generalizability, and implementation

The clinical value of a prediction tool depends on external validation, calibration, transportability, and implementability, dimensions that were inconsistently addressed in the included studies. Calibration, transportability, and the handling of missing or operator-dependent variables were seldom reported, and few studies measured implementation endpoints such as time to plasma-exchange initiation or avoidance of unnecessary plasma exchange. Future evaluations should report calibration and decision-analytic metrics, prespecify missing-data handling, and measure workflow integration alongside diagnostic accuracy.

### 5.4 Clinical implications

Because TTP's nonspecific presentation mimics other TMAs, rapid and reliable ADAMTS13 testing lets treatment begin sooner, and hospital-based testing reduces dependence on external laboratories. High-specificity tools are equally important when treatment carries its own risks and significant costs.

When ADAMTS13 activity is borderline or clinical features are atypical, combining assay results with platelet count, LDH, creatinine, reticulocyte percentage, and clinical scores can improve triage and reduce both under-treatment and over-treatment (50).

### 5.5 Comparison with existing literature

Our synthesis aligns with existing literature while showing how these assays can be combined in practice. Severe ADAMTS13 deficiency below 10 IU/dL is highly specific for TTP and remains central to diagnosis. A recent meta-analysis of over 4,000 samples (Deshpande et al., 2025) (52) reported that automated chemiluminescent ADAMTS13 assays achieve approximately 0.98 sensitivity and 0.99 specificity, with excellent negative predictive value and strong correlation with FRETS-VWF73. Nevertheless, Papakonstantinou et al. (17) highlight that ADAMTS13 activity alone may not suffice for equivocal cases, noting that diagnostic pathways are increasingly improved by integrating clinical scores and adjunct biomarkers alongside laboratory results.

ADAMTS13 activity alone is insufficient for diagnosis, and the field is moving toward automated, standardized, in-hospital testing (33).

An important interpretive caveat is the distinction between analytical and clinical validation. Several assays here, including CLEIA, fibre-optic surface plasmon resonance, and the open-conformation ELISA, are supported chiefly by analytical agreement with reference methods, which shows measurement concordance but not clinical performance; fewer, such as HemosIL AcuStar on consecutive samples and Technoscreen in suspected-TMA cohorts, provide patient-level validation.

## 6 Limitations

Several limitations should be considered when interpreting these findings. First, the total number of studies included is relatively small, and substantial heterogeneity exists across study designs, patient populations, assay methodologies, thresholds, sample handling, and reported outcomes. This variation precludes formal meta-analysis and limits direct, standardized comparisons across platforms, thereby reducing confidence in any definitive ranking of one system over another. Furthermore, the existing evidence relies predominantly on observational data; robust diagnostic accuracy trials and randomized comparisons of specific diagnostic strategies remain lacking. Finally, comparisons between algorithms are confounded by their variable configurations and their dependence on local workflows and case mix.

Several studies also used case-control or two-gate designs, recruiting pre-confirmed iTTP patients alongside healthy donors rather than undifferentiated all-comers, which likely overestimated performance (5, 33, 37); banked samples pre-selected for activity range further limit applicability to real-time emergency triage.

These selected designs are prone to spectrum bias, so the highest reported accuracy figures are best read as analytical proof-of-concept rather than validated clinical accuracy. Our exclusion of conference abstracts may also under-capture recent tools such as the externally validated model TTP-14 (53); the three clinical prediction tools synthesised here are therefore not the complete contemporary landscape.

Publication bias is also a concern, since studies with favorable results are more likely to be published than those with inconclusive findings.

### 6.1 Future research directions

Future work should prioritize rigorous multicentre validation in diverse, undifferentiated suspected-TMA populations, using standardized ADAMTS13 measurement and reporting. Prospective and high-quality retrospective multicentre cohorts, external-validation and implementation studies, and pragmatic evaluations are all valuable, as is further validation of near-patient testing in emergency and resource-limited settings, with attention to clinical impact and cost-effectiveness.

## 7 Conclusion

Advances in TTP diagnostics have improved the speed of identifying severe ADAMTS13 deficiency without sacrificing reliability. Rapid automated chemiluminescent and fluorescence platforms now offer turnaround times compatible with the urgency of suspected TTP, with strong concordance with reference standards and high sensitivity and specificity in acute settings. Adjunct biomarkers, inhibitory antibody testing, and conformation assays may further improve precision in borderline or ambiguous cases. TTP diagnosis is becoming faster and more integrated, and the strongest pathways combine several parameters to support earlier treatment decisions and better risk stratification. Modern ADAMTS13-based diagnostics are analytically strong and increasingly usable at the point of care when they matter most.

## Data Availability

The original contributions presented in the study are included in the article/Supplementary Material, further inquiries can be directed to the corresponding author/s.
